# Hypothesis and Theory: A Pathophysiological Concept of Stroke-Induced Acute Phase Response and Increased Intestinal Permeability Leading to Secondary Brain Damage

**DOI:** 10.3389/fnins.2020.00272

**Published:** 2020-04-21

**Authors:** Fabienne Ferrara, Vilia Zeisig, Sören Pietsch, Rita Rütten, Antje Y. Dreyer, Laura Pieper, Ann-Kathrin Schatzl, Damian D. McLeod, Henryk Barthel, Johannes Boltze, Wieland Schrödl, Björn Nitzsche

**Affiliations:** ^1^Fraunhofer Institute for Cell Therapy and Immunology (IZI), Leipzig, Germany; ^2^Clinic and Policlinic for Nuclear Medicine, University of Leipzig, Leipzig, Germany; ^3^Klinik und Poliklinik für Kinder und Jugendmedizin, Universitätsklinikum Leipzig, Leipzig, Germany; ^4^Institut für Veterinär-Epidemiologie und Biometrie, Freie Universität Berlin, Berlin, Germany; ^5^OncoRay - National Center for Radiation Research in Oncology, Faculty of Medicine and University Hospital CG Carus, TU Dresden, HZDR, Dresden, Germany; ^6^School of Biomedical Sciences and Pharmacy, Faculty of Health and Medicine, and Hunter Medical Research Institute, The University of Newcastle, Callaghan, NSW, Australia; ^7^School of Life Sciences, Faculty of Science, University of Warwick, Coventry, United Kingdom; ^8^Faculty of Veterinary Medicine, Institute of Bacteriology and Mycology, University of Leipzig, Leipzig, Germany

**Keywords:** ischemic stroke, immune response, intestinal permeability, acute phase proteins, systemic inflammation, large animal stroke models, cerebral ischemia, gut-brain axis

## Abstract

Gut integrity impairment leading to increased intestinal permeability (IP) is hypothesized to be a trigger of critically illness. Approximately 15–20% of human ischemic stroke (IS) victims require intensive care, including patients with impaired level of consciousness or a high risk for developing life-threatening cerebral edema. Local and systemic inflammatory reactions are a major component of the IS pathophysiology and can significantly aggravate brain tissue damage. Intracerebral inflammatory processes following IS have been well studied. Until now, less is known about systemic inflammatory responses and IS consequences apart from a frequently observed post-IS immunosuppression. Here, we provide a hypothesis of a crosstalk between systemic acute phase response (APR), IP and potential secondary brain damage during acute and subacute IS stages supported by preliminary experimental data. Alterations of the acute phase proteins (APPs) C-reactive protein and lipopolysaccharide-binding protein and serum level changes of antibodies directed against *Escherichia coli*-cell extract antigen (IgA-, IgM-, and IgG-anti-*E. coli*) were investigated at 1, 2, and 7 days following IS in ten male sheep. We found an increase of both APPs as well as a decrease of all anti-*E. coli* antibodies within 48 h following IS. This may indicate an early systemic APR and increased IP, and underlines the importance of the increasingly recognized gut-brain axis and of intestinal antigen release for systemic immune responses in acute and subacute stroke stages.

## Introduction

Gut integrity (GI) plays an important role in balancing permeable gut functions required for the uptake of nutritional components versus preventive functions such as forming a barrier against pathogen egress (Doig et al., 1998; Otani and Coopersmith, 2019). GI impairment and subsequently increased intestinal permeability (IP) are believed to be a major pathophysiological elements in a number of severe conditions. They have been described in chronic heart failure (Doig et al., 1998; Sandek et al., 2014), complications in intensive care unit (ICU) patients (Klehmet et al., 2009) and in individuals undergoing cardiopulmonary bypass (Riddington et al., 1996), as well as after traumatic brain injury (Bansal et al., 2009). GI impairment and increased IP are supposed to be related to inadequate mucosal perfusion (Doig et al., 1998; Klehmet et al., 2009; Sandek et al., 2014) or a potential epithelial disruption by proinflammatory cytokines such as TNF-α (Triantafilou and Triantafilou, 2002). Decreased intestinal blood flow and increased proliferation of mucosal bacteria were correlated with a higher level of systemic anti-lipopolysaccharide (LPS) IgA in patients with critical chronic heart failure (Sandek et al., 2014).

Despite the recent advances in acute ischemic stroke (IS) management and care (Saver et al., 2016), IS is still a leading cause of chronic disability and death (Zazulia, 2009). Next to primary ischemic and secondary neuroinflammatory brain damage, IS patients also suffer from systemic stroke sequelae such as stroke-induced systemic immune suppression (SIIS) leading to pneumonia or severe gut alterations including dysmotility, microbiotic dysbiosis, and bleedings (Arya and Hu, 2018). While SIIS and related pulmonary infections have been investigated decently (Prass et al., 2003; Dirnagl et al., 2007; Klehmet et al., 2009), not much is known about intestinal epithelial barrier dysfunction following IS. Since GI and increased IP could serve as an important systemic immunological and inflammation trigger, they have recently been discussed as potential elements of systemic IS pathophysiology and as potential therapeutic targets (Zazulia, 2009; Wen and Wong, 2017). Based on a set of preliminary experimental data, we hypothesize that an impaired GI with increased IP can emerge after IS and could potentially pave the way for endogenous gut-borne infections, or fuel reciprocal immune responses leading to secondary brain damage.

## Intestinal Permeability and Stroke Models

Recent investigations on the role of the microbiome in mouse IS models suggested a protective function of bacteria in the conventional gut flora as intestinal dysbiosis was associated with poor outcome (Winek et al., 2016; Sadler et al., 2017). However, rodent data on disturbed GI and increased IP following IS are controversial. Some studies report bacterial translocation and sepsis after IS in rats and aged mice (Crasper et al., 2016), while others did not find evidence for increased IP and bacterial translocation 3 days after transient middle cerebral artery occlusion (Oyama et al., 2018). This may raise the question whether these findings from rodent IS models might be breed-, supplier- or even model-specific.

The Stroke Treatment Academic Industry Roundtable (STAIR) expert consortium recommends the additional use of suitable gyrencephalic models of focal cerebral ischemia to increase the validity of experimental findings (Fisher et al., 2009). Due to a closer similarity to humans regarding neuroanatomical and physiological features, large animal models are believed to mimic the clinical situation of human stroke patients realistically (Dirnagl et al., 2013). In particular, large animals might be useful to investigate GI breakdown and increased IP after IS. Ruminants, for which IS models are available (Boltze et al., 2008; Wells et al., 2012), possess a much larger gastrointestinal tract than rodents and humans in both absolute and relative terms, and a higher physiological bacteria load, what may be an advantage when aiming to detect potential systemic immunological consequences.

## Hypothesis: Stroke-Induced Acute Phase Response and Increased Intestinal Permeability Lead to Secondary Brain Damage

The mechanism behind a potential GI impairment and increased IP after IS remain poorly understood. Hypothetically, impaired GI and increased IP can be caused by inadequate mucosal perfusion or epithelial breakdown mediated by proinflammatory cytokines such as TNF-α or zonulin. This has been described in the context of ischemic diseases and reduced organ motility (Rahman et al., 2018). Indeed, inflammatory cytokines can compromise intestinal mucosa integrity by affecting endothelial (gate) and tight junction (fence) functions (Bruewer et al., 2003).

**FIGURE 1:**
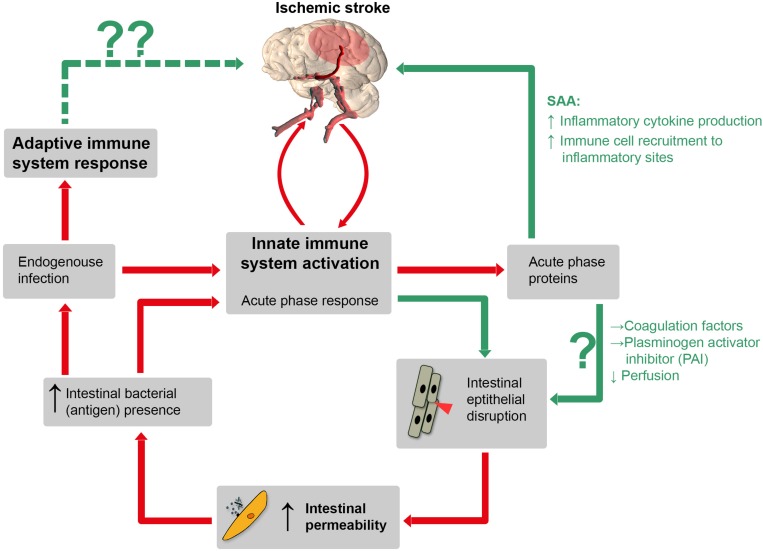
Schematic illustration of a hypothetical pathophysiological cascade following ischemic stroke (IS): the innate immune system is activated by cerebral ischemia due to release of inflammatory cytokines after blood-barrier-breakdown. Hence, these cytokines could directly inflict “off-site” damage to the intestinal mucosa and induce the production of APPs such as serum amyloid A (SAA), coagulation factor and plasminogen activator inhibitor (PAI). SAA itself stimulates further inflammatory cytokine production via positive feedback stimulation. Other APPs, such as coagulation factors and PAI can impair capillary perfusion and, importantly, increase IP which is associated with bacterial translocation. Consequently, both pathways increase the amount of pro-inflammatory mediators, which leads to a prolonged inflammatory stage following IS. Red lines: established mechanisms, green lines: potential mechanisms (according to our hypothesis). Whether or not adaptive immune processes, augmented by the translocation of bacteria and bacterial antigens from the gut, can inflict secondary brain damage is entirely speculative (dotted green line, upper left).

Within minutes, IS leads to a four-step process of ischemia-related blood-brain barrier breakdown (BBB) in peri-infarct regions (Ballabh et al., 2004; Sandoval and Witt, 2008; Krueger et al., 2015), accompanied by endothelial cell damage and loss (Krueger et al., 2015). BBB disruption then facilitates exchange of blood components and brain antigens such as brain myelin basic protein (del Zoppo and Mabuchi, 2003; Offner et al., 2006; Krueger et al., 2015). Inflammatory cytokines are released into the circulation in large amounts after IS and hence could inflict “off-site” damage to the intestinal mucosa. Moreover, inflammatory cytokines induce the production of acute phase proteins (APPs) in the liver, but interestingly also in intestinal epithelium (Molmenti et al., 1993; Wang et al., 1998). Some APPs, such as serum amyloid A (SAA) stimulate inflammatory cytokine production themselves, providing a positive feedback mechanism. SAA, once present in the circulation, also increases the recruitment of immune cells to inflammatory sites. Other APPs, such as coagulation factors and plasminogen activator-inhibitor foster coagulation and might negatively affect perfusion, contributing to thromboinflammation in cerebral and intestinal capillaries. A vicious circle finally leading to increased secondary brain damage might evolve (Figure 1).

## Statement of Hypothesis

### Collection of Preliminary Data to Support the Hypothesis

In order to find support for abovementioned hypothesis, we took advantage of a running experiment in an ovine IS model in which peripheral blood samples were collected at days 1, 2, and 7 following IS. We focused on signs for potential (i) acute phase response (APR), (ii) IP alterations, and (iii) interaction between intestinal antigen translocation and systemic inflammation. Specifically, we determined levels of the APPs C-reactive protein (CRP), lipopolysaccharide-binding protein (LBP), and of and IgA-, IgM-, and IgG-anti-*E. coli* antibodies 1, 2, and 7d following IS. *E. coli* belongs to the physiological adhesive intestinal flora in sheep (Wang et al., 1998), but does not egress from the intestine in the steady state. Hence, changes of IgA-, IgM-, and IgG-anti-*E. coli* antibody levels could reflect IP alterations following IS. Animals were subjected to permanent middle cerebral artery occlusion (pMCAO) as described elsewhere (Boltze et al., 2008). Please refer to the Supplementary Material for methodological details.

All animals were considered clinically healthy prior to surgery (for further details see Supplementary Tables S1 and Supplementary Tables S2). Animal health was assessed one day after pMCAO and every second day thereafter. No clinical signs of infection (e.g., fever) were observed. A standardized anesthesia and medication scheme was performed in all animals (see Supplementary Table S3). However, two animals with massive strokes were sacrificed 1d post IS due to severe neurological dysfunction and rapid worsening of general condition. These animals were excluded from further analysis. Stroke induction failed in one animal (no IS at day 1). Since this particular animal underwent the same procedure as other pMCAO animals, it was kept and served as a sham reference to control for surgery-related effects in APR.

### Findings

#### Imaging Findings

Combined positron-emission tomography and magnetic resonance (PET/MR) imaging confirmed a perfusion deficit corresponding to the ischemic area within the MCA territory at day 1 after pMCAO in all but the sham reference animal (Supplementary Figure SF1B). Details on the lesion and lesion size (t2w TSE defect volume in ml) at days 1 and 7 post pMCAO are given in Supplementary Figure SF1 and Supplementary Table S4.

#### Systemic Acute Phase Protein Levels

**FIGURE 2:**
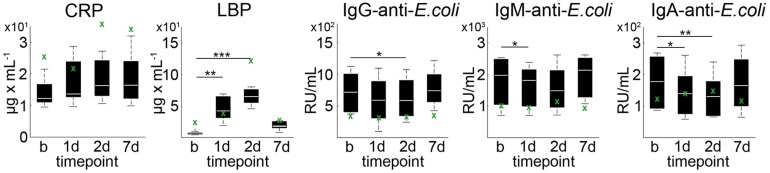
Acute phase proteins and anti-*E. coli* antibodies in plasma. CRP, LBP as well as IgA-, IgM- and IgG-anti-*E. coli* antibodies exhibited a high inter-individual variance at baseline (b) and 1, 2, and 7 days post pMCAO. Boxplots indicate the mean at each time point (white bar), 1st and 3rd quartiles, and 95% confidence intervals. Significance level of paired two-sample *t*-test against baseline were *p* < 0.05 (*), *p* < 0.01 (**), and *p* < 0.001 (***). The green crosses (x) indicate sole data points from the animal with failed pMCAO (no stroke at day 1) serving as a sham reference for surgery-related effects.

**FIGURE 3:**
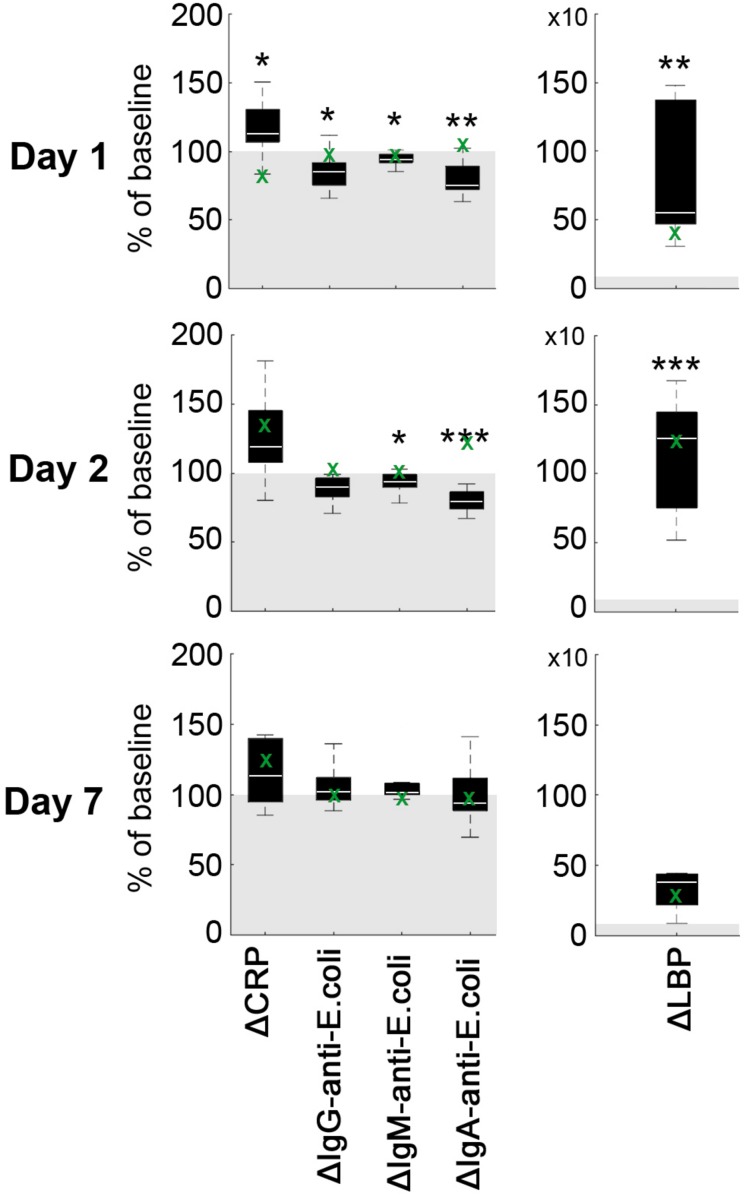
Difference of acute phase proteins ΔCRP and ΔLBP as well as IgA-, IgM- and IgG-anti-*E.coli* antibodies. Values are given as percentage of baseline (gray area). Boxplots indicate the mean difference at each time point (white bar), 1st and 3rd quartiles, and 95% confidence intervals. Significance level of one-sample *t*-test against 100 (baseline) is indicated by *p* < 0.05 (*), *p* < 0.01 (**) and *p* < 0.001 (***). The green crosses (x) indicate sole data points from an animal with failed pMCAO (no stroke at day 1) serving as a sham reference for surgery-related effects.

Average CRP plasma levels tended to increase on day 1 after pMCAO as compared to baseline (*p* = 0.067), but not on day 2 (*p* = 0.213) and day 7 (*p* = 0.241, Figure 2). Statistically significantly increased levels of mean LBP were detected on day 1 (*p* = 0.002) and day 2 (*p* < 0.001) when compared to baseline (Figure 2). At each time point (baseline/1d/2d/7d), a relatively high inter-individual variability could be detected for CRP (39.6%/41.3%/35.7%/43.2%) and LBP (39.5%/41.7%/19.7%/101.5%). Baseline-corrected values showed a significant increase of CRP on day 1 (*p* = 0.028), while LBP was increased on day 1 (*p* = 0.002) and 2 (*p* < 0.001; all Figure 3). Baseline APP values in the sham reference animal were higher than in other subjects, but followed a similar kinetic except for a slight decrease of CRP values at d1 (Figure 2, green crosses).

#### IgA-, IgM-, and IgG-Anti-*Escherichia coli* Antibodies

Average plasma levels of IgA-, IgM-, and IgG-anti-*E. coli* antibodies decreased within the first 2 days after pMCAO, followed by an minor increase on day 7 (Figure 3). In detail, mean IgG-anti-*E. coli* antibody levels were significantly decreased on day 2 (*p* = 0.0012), and a significant (*p* = 0.048) drop of IgM-anti-*E. coli* antibodies levels was observed on day 1 post pMCAO. Additionally, IgA-anti-*E. coli* antibodies were significantly decreased at day 1 (*p* = 0.006) and day 2 (*p* = 0.001). Since the coefficient of variation for IgG-anti-*E. coli* (41.3%/55.7%/48.1%/35.4%), IgM-anti-*E. coli* (42.2%/40.0%/45.9%/33.7%) and IgA-anti-*E. coli* (42.8%/48.5%/49.7%/48.6%) was high at each time point, baseline-corrected values were analyzed (Figure 3). Values relative to baseline for IgG-anti-*E. coli* significantly decreased on day 1 (*p* = 0.048), and those for IgM- and IgA-anti-*E.coli* on days 1 (*p* = 0.008, *p* = 0.002) and 2 (*p* = 0.045, *p* < 0.001) post pMCAO (Figure 3). Antibody levels remained almost constant in the sham reference samples, apart from an isolated increase of IgA-anti-*E.coli* on day 2.

#### Correlation With Ischemic Brain Damage

A negative correlation (*r* = −0.700, *p* = 0.036) was found between LBP and IgA-anti-*E. coli* values 1d post pMCAO. Moreover, the levels of IgM-anti-*E. coli* correlated positively with rCBF values (*r* = 0.927, *p* = 0.003) 1d after pMCAO. In addition, a negative correlation (*r* = -0.901, *p* = 0.014) was detected between total brain volume (excluding IS lesion) 7d following pMCAO and IgG-anti-*E. coli* 2d post pMCAO. We found no further significant correlations between any measured parameters. Detailed results of all correlation analyses are given in Supplementary Tables S5–S8.

### APP Response Following IS

CRP and LBP are relevant APPs in humans (Schumann and Zweigner, 1999) and sheep (Schroedl et al., 2001; Ulutas and Ozpinar, 2006). In fact, clinical investigations revealed that blood-borne markers of peripheral inflammation, including increased CRP, IL-6 or white cell counts, are present within the first week following IS in human patients, thus providing evidence of early and sustained inflammatory conditions (Emsley et al., 2003). Elevated CRP levels in cardiovascular ischemia are hypothesized to increase myocardial damage in the infarct area, likely by activating the complement cascade (Nijmeijer et al., 2003). Additional myocardial damage can in turn increase CRP levels, forming a part of the rational basis for our hypothesis that a similar mechanism might exist in stroke. This vicious circle is thought to contribute to poor outcome (Gill et al., 2004; Pepys et al., 2006; Slagman et al., 2011). CRP is normally absent from serum, but typically rises within 6 h of inflammation onset in humans with serum levels increasing up to 1000-fold within 24 h in cases of severe inflammation (Roudbary et al., 2011). LBP plays an essential role for the innate immune response against bacterial infection (Triantafilou and Triantafilou, 2002; Worthmann et al., 2015) and is capable of binding bacterial LPS. It activates macrophages and monocytes via CD14 and toll-like receptors (TLR), leading to release of pro-inflammatory cytokines (Schumann and Zweigner, 1999). In healthy humans, LBP serum values fluctuate between 5–10 μg/ml, and can increase up to 10-fold during the acute stage of severe inflammatory processes (Myc et al., 1997). LBP mean levels in our data set increased from 6 up to 47 μg/ml at day 1 after pMCAO, quantitatively resembling the situation in humans. LBP is usually produced in the liver, but can also be produced extrahepatically under pro-inflammatory conditions. For instance, LBP production was observed in murine and human intestinal epithelium in cases of severe systemic inflammation (Vreugdenhil et al., 1999, 2000). High amounts of ruminant LBP is also expressed within the forestomach, small intestine crypts and colon glands, at which it likely plays an important role in mucosal innate pathogen defense (Rahman et al., 2010). The data may further indicate an early increase of local gastrointestinal LBP production in sheep under pro-inflammatory conditions as previously assumed (Rahman et al., 2010). This might indicate a systemic APR in the early stage of experimental IS in sheep, thus supporting the reference of IS-triggered cascade of systemic immunoreactions observed in rodents (Offner et al., 2006) and humans (Emsley et al., 2003).

### Connecting APP Response, IP, and Secondary Brain Damage

Ruminants possess a much larger gastrointestinal tract than rodents and humans. The continuous physiological presence of bacteria such as *E. coli* ensures that a “baseline” antibody titer is maintained in the sheep blood. Interestingly, levels of all tested anti-*E. coli* antibodies were reduced during the first 48 h post IS, but recovered to baseline levels 7d post IS. Theoretically, decreased antibody levels may be related to changes of intestinal barrier integrity. We hypothesize that the decreased antibody levels are related to a suddenly increased presence of bacterial antigens in plasma, as a result of translocation through the gut wall, thus leading to initial anti-*E. coli* antibody consumption. Our hypothesis is underpinned by the simultaneously increased LBP levels as well as by a negative correlation between LBP and anti-*E. coli* antibodies within 24 h post IS. Local LBP expression might be increased by pro-inflammatory factors such as LPS (Su et al., 1994; Dentener et al., 2000; Bruewer et al., 2003). However, our results could also be related to SIIS in general. Hence, to substantiate our hypothesis, further investigations should be performed, including measurement of cytokine (e.g., TNF-α) as well as measurements of the sympathetic nervous system status (e.g., catecholamine and cortisol), in order to explore the entire inflammatory and immunosuppression process.

Enhanced IP may enable antigen fluxes through the intestinal wall or could activate the local intestinal immune system. Both events could increase the amount of inflammatory mediators such as intestinal factors including *i-*PAMPs (pathogen associated molecular pattern, e.g., *E. coli* lipopolysaccharide: F-, K-, or H-antigens) F-, K-, or H-antigens or cytokines within the circulation. In summary, increased IP could hypothetically lead to a prolonged inflammatory stage following IS, which may further aggravate IS outcome. We found some indirect indication for a potential relationship between increased IP and a systemic immune reaction. We detected that higher free anti-*E. coli* antibody levels at day 2 were related to smaller lesions (rCBF deficits) 1d following IS (see Supplementary Table S8). In turn, higher levels of IgA-anti-*E. coli* (2d post IS) are related to reduced post-stroke total brain volume (indicating post-IS tissue loss) 7d following IS (see Supplementary Table S8). This may imply that increased IP could amplify or modify systemic-adaptive and- innate immune reaction patterns, cumulating in secondary brain damage. Subsequent studies must be performed to investigate this assumption, e.g., determination of bacterial antigen, cytokine or endotoxin measurements, histological assessment and CFU content determination in blood, gut lymph nodes, spleen, liver, and lung.

### Critical Appraisal of Preliminary Data and Hypothesis

Rodent brain structure and size, genetic homogeneity, and differences in immunobiology may limit the translation of experimental findings from rodent models to the human patients. Large animal IS models are potentially useful for IP investigations following IS. However, outcome variability is often higher than in rodent models (Herrmann et al., 2019), what may reflect the inter-individual variations observed in human stroke patients. Accordingly, we found a relatively high inter-individual variation for all measured blood parameters, but overall data indeed indicate increased IP after IS.

A limitation of the supporting data is the small statistical sample size and the existence of only one sham case. In consequence, general effects of the surgical procedure, anesthesia or medication on evaluated blood markers cannot be excluded. The responses of CRP and LBP in the sham subject with identical surgical intervention except for MCA occlusion show a relative similar pattern than that in other animals, apart from a drop in CRP value at day 1 (Figure 2). These APPs would be expected to generally increase after tissue trauma (Baumann and Gauldie, 1994; Nijmeijer et al., 2003) as for instance exerted by the invasive MCAO model (trepanation). However, antibody titers relative to baseline remained almost constant in this animal over the course of the experiment. This makes it less likely that the changes observed in the other subjects are simply caused by the surgical intervention itself, supporting our hypothesis.

Potentially, there might be an interaction between medication and post-stroke immune system responses, i.e., clinical signs of infection following pMCAO could be masked by medication. However, there is no evidence of relevant impact of butorphanol-, cyclooxigenase inhibitor flunixin-, or antibiotic treatment (enrofloxacin) on CRP and LBP levels in contemporary literature (Löscher et al., 2014; Henke et al., 2015). However, systemic antibiotic therapy was given in a uniform scheme as required by the experimental protocol of the study these cases were derived from. This may have counteracted some of the consequences of potential *E. coli* traffic across intestinal barriers and on inflammatory response, but post-stroke changes in antibody and APP levels were nevertheless large enough to be statistically significant despite the small sample sizes.

## Conclusion

We initially characterized the modification of the APR and the interactions between the systemic immune system and IP following IS in an ovine IS model. Our findings confirm the reaction patterns of CRP and LBP as APPs following IS, and revealed preliminary indications for alterations of intestinal barrier functions. In addition, we found indirect indications of IP enhancement in the early stage of IS in sheep. To our knowledge, this was first explored in a large animal model. Further confirmatory investigations, e.g., direct identification of bacterial antigens, endotoxin measurements, histological assessment and colony forming unit (CFU) determination in blood, gut lymph nodes, spleen, liver, or lung, need to be performed. Measurements of inflammatory cytokines as well as potential interaction with the sympathetic nervous system (e.g., catecholamine and cortisol determination) should be conducted to gain a more holistic picture of the entire pathophysiological cascade. Moreover, any effect of antibiotic treatment should be excluded by a carefully planned study design.

## Data Availability Statement

All raw data are available upon reasonable request.

## Ethics Statement

The animal study was reviewed and approved by the Experimental Animal Committee of the Regional Council of Leipzig, Germany (animal protocol number 09-11).

## Author Contributions

FF designed the study, performed the experiments, obtained and analyzed the data, drafted, and reviewed the manuscript. VZ obtained and analyzed the data and reviewed the manuscript. SP helped in performing the experiments and the obtained data. RR and DM performed the experiments and reviewed the manuscript. AD performed the experiments and obtained the data. LP analyzed the data. A-KS helped in performing the experiments. HB obtained and analyzed the data. JB analyzed the data, drafted, and reviewed the manuscript. WS analyzed the data and reviewed the manuscript. BN obtained and analyzed the data, drafted, and reviewed the manuscript. All authors acknowledged the final version of the manuscript.

## Conflict of Interest

The authors declare that the research was conducted in the absence of any commercial or financial relationships that could be construed as a potential conflict of interest.
