# Supplementary material for: Hypothesis and Theory: A Pathophysiological Concept of Stroke-Induced Acute Phase Response and Increased Intestinal Permeability Leading to Secondary Brain Damage
Source: Front Neurosci. 2020 Apr 21;14:272. doi: 10.3389/fnins.2020.00272 (PMC7186394; doi:10.3389/fnins.2020.00272)
Supplement: Supplementary file 1 [file Presentation_1.pdf]

Supplements

Material and Methods

Experimental animals: Ten healthy hornless outbreed Merino rams (12 months old, 50-70 kg) were used. Animals were kept in small flocks with *ad libitum* access to food and water. Food, but not water was withdrawn 12h prior to surgery. Except for day of surgery, outdoor browsing was allowed for 5 hours a day. Skilled veterinarians assessed animals' general health condition 3 days prior to and at the day of surgery, one day thereafter and on demand. Health assessments included clinical examination and blood screening (for further details see Supplementary Tables S1 and S2). The study design is given in Fig. 2. The present investigations on systemic immune cascade and IP alterations following IS were carried out as part of an ongoing main study. Due to the legal requirement to implement the 3R principle (Replace, Reduce and Refine) a sham group for the main study was not approved by the responsible authority.

Surgical procedure: Surgery was performed under controlled mechanical ventilation and general anesthesia (Draeger Primus Plus, Lübeck, Germany) as described previously [1]. Animals were continuously monitored (electrocardiogram, arterial blood pressure, oxygen saturation, expiratory carbon dioxide content, and body temperature) during the entire surgical procedure (Infinity Gamma XXL, Draeger, Lübeck, Germany). Following trepanation, dura incision, and exposition of the left middle cerebral artery (MCA), permanent MCA occlusion (pMCAO) was induced by electrocoagulation (ME 411, KLS Martin, Freiburg, Germany). Trepanation was covered with the temporalis muscle, followed by subcutaneous and skin sutures using 2-0 absorbable filaments (Ethicon Ltd, Norderstedt, Germany). Animals remained intubated under permanent monitoring until the swallowing reflex was restored. A standardized anaesthesia and medication scheme was performed in all animals (see Supplementary Table S3). Animals were thoroughly monitored until they were able to stand and move. Subsequently, food and water was provided, and animals were allowed to recover.

Blood sample analyses: Blood samples (heparinized plasma) were taken prior to surgery (baseline) and on day 1, 2, and 7 after pMCAO (see Fig. 2) to investigate APR and potential antibody titers. Blood samples were centrifuged (2000 x g, 4°C) for 10 min and plasma was stored at -20°C. Levels of C-reactive protein (CRP) and lipopolysaccharide-binding protein (LBP) were determined by two-site (sandwich) enzyme-linked immunosorbent assay (ELISA) [2, 3] or, in case of IgA-, IgM- and IgG-anti-*E. coli* antibodies, by an indirect ELISA [3]. Note, that only the steps varying from those previously published are described below.

<u>*Common ELISA steps:*</u> Concentrations were determined by optical density (OD) using a microplate-ELISA-reader (type anthos htIII) at 450 nm. For all ELISAs, the intra-assay variation was <10 %, while inter-assay variation was <15 %. All ELISAs were performed on ELISA-plates (96-well, high binding, Costar®, Sigma Aldrich, St. Louis, Missouri, USA). All plates were incubated on a microtiter plate shaker (500 rpm) at room temperature for 1h, applying a volume of 100 µl per well. 0.1 M NaHCO3 was used as the coating buffer for CRP and LBP and 0.9 % NaCl-solution for the anti-LPS (*E. coli*) antibodies. The washing buffer was 0.9 % NaCl in aqua dest. containing 0.1 % (vol/vol) Tween 20. 3 mM H_2O_2, and 1 mM 3.3',5.5'-tetramethylbenzidine in 0.2 M citrate buffer (pH 3.95) was used as the colorimetric

substrate. The substrate reaction was stopped with 50 µl/well 1 M H_2SO_4. All chemicals were purchased from the same supplier (Sigma-Aldrich Chemie GmbH, Taufkirchen, Germany). The standard curve (standard-concentrations vs. OD-values) was determined using TableCurve software (Systat Software, Erkrath, Germany) and the concentrations of CRP, LBP or IgA-, IgM- and IgG-anti-*E. coli* antibodies were then calculated.

Two-site (sandwich) ELISA for CRP measurement: The CRP concentration was determined by ELISA as described previously [3]. In brief, the CRP-concentration was measured with purified ovine CRP as standard (calcium dependent phosphorylcholin-affinity chromatography and ion exchange chromatography with DEAE-Sepharose). IgG-anti-human CRP (DAKO, 1:2000) was used as coating antibody with cross-reactive anti-ovine CRP antibodies. Pooled ovine blood plasma served as a reference (lab-internal reference plasma, previously derived from healthy sheep). The standard concentration ranged from 1 to 100 ng/ml. The samples were diluted 1:1000. Phosphate-buffered saline (PBS, pH 7.35) containing 0.1% Tween 20 (vol/vol) was used as assay buffer for dilution of the standard and plasma samples. IgG-anti-human CRP conjugated with horseradish peroxidase (POD, DAKO, 1:2000) was used as the detection antibody. Before measurement, ovine CRP was purified as described elsewhere [4]. In brief, ovine serum was purified and centrifuged at 3000 x g for 30 min and kept at -20°C. The purified serum was diluted with the same volume of Dulbecco's PBS. The precipitate was removed and ammonium sulfate (75% saturation) was added to the supernatant. The final precipitate was then dissolved in 0.01 M Tris buffer (pH 8.0), containing 0.14 M NaCl, 0.002 M CaCl2, and 0.01% NaNi (Tris-Ca buffer), and dialyzed against the same buffer. Thereafter, the dialyzed sample was centrifuged and the supernatant was exposed to a 4.5 x 30 cm column packed with 4% Seakem HE-agarose gel particle (FMC Co. Ltd., Rockland, ME, USA). The supernatant was fragmented through a 140 pm mesh and equilibrated with Tris-Ca buffer. Following washing with the same buffer (1.5 I), bounded proteins were eluted with the buffer containing 0.01 M EDTA (Tris-EDTA buffer), and fractionated into 6 ml samples. The protein fractions were pooled dialyzing against 0.02 M Tris buffer pH 7.4) containing 0.04 M NaCl and 0.001 M EDTA. Then, the prepared protein fractions were concentrated by ultrafiltration. Approximately 21.8 mg protein (0.7 ml) was applied to a 75 x 7.5 mm DEAE-SPW HPLC column (Toxosoda, Tokyo, Japan) equilibrated with the starting buffer. The standard concentration ranged from 1 to 100 ng/ml. The samples were diluted 1:2000 and Dulbecco-PBS (pH 7.35) with 0.1% (vol/vol) Tween 20 was used as buffer.

Two-site (sandwich) ELISA for LBP measurement: ELISA-plates were coated with 1 µg/ml monoclonal anti-LBP-antibody (mouse IgG1, big 48, biometec, Greifswald, Germany). Pooled blood plasma from healthy sheep (see above) served as the standard. The ovine LBP-concentration in an ovine standard serum was calibrated by affinity-purified bovine LBP (LPS-affinity chromatography and ion exchange chromatography with Q Sepharose XL). The blood samples were diluted 1:1000 or higher, and the standard range was between 3 and 200 ng/ml of ovine LBP. 50 mM Tris-HCl (pH 7.35) with 0.15 M NaCl, 0.1% TopBlock (Fluka, Buchs, Switzerland) and 0.1% Tween 20 served as dilution buffer. A monoclonal anti-LBP-mouse IgG1 (big 412 with POD; biometec, Greifswald, Germany), diluted in assay buffer to a concentration of 0.1 µg/ml, was used for detection.

Indirect-ELISA for IgA-, IgM- and IgG-anti-E. coli antibody measurement: ELISA-plates were coated with 100 µl/well of 2 µg/ml E. coli-extracted antigen in 0.15 M NaCl dissolved in aqua ultra pure (Biochrom, Berlin, Germany). The antigen was extracted from eight *E. coli* strains according to manufacturer's instructions by using B-PER-bacterial protein extraction reagent (Thermo Scientific, Rockford, USA). After incubation for antigen coating over night at 4-6°C, the plates were washed twice with 0.9% NaCl-solution, before 150 µl of 1% bovine casein in a 0.9% NaCl solution were added per well. After 30 minutes of incubation, 50 µl of

the plasma samples diluted in 4-fold PBS (Dulbecco without Ca2+ and Mg2+, pH 7.35) containing 0.4% of Tween 20 at 1:50 (for detection of IgG), 1:100 (for IgM), and 1:20 (for IgA) were added to the wells. Various dilutions of ovine internal lab reference plasma (see above) served as the laboratory standard. The internal definition of the antibody standard was 100 relative units per millilitre (RU/ml). After incubation and three washing steps (see above), the following conjugates were added: POD-conjugated IgG (rabbit) anti-ovine IgG (diluted 1:5000, Bethyl Laboratories Inc., Montgomery, USA), POD-conjugated IgG (rabbit) anti-ovine IgM (1:10000, Bethyl Laboratories Inc., Montgomery, USA), and POD-conjugated IgG (rabbit) anti-ovine IgA (1:2000, Bethyl Laboratories Inc., Montgomery, USA). Antibodies were solved in PBS (Dulbecco, pH 7.35) containing 0.1% of bovine casein and 0.1% Tween 20. POD activity was measured after incubation and three washing steps as described above, and RU/ml were calculated for each plasma sample in relation to the internal antibody standard.

Imaging procedure: Lesion volume and cerebral perfusion deficit were evaluated by combined, simultaneous positron emission and magnet resonance tomography (PET/MR; 3T Biograph mMR, Siemens Healthcare, Erlangen, Germany) using a $[^{15}O]H_2O$ scan and different MR sequences (see below) simultaneously (3T Biograph mMR, Siemens Healthcare, Erlangen, Germany) 1d following pMCAO. A follow-up MRI was performed on day 7 after pMCAO using a clinical 1.5T MRI (Philips Medical System). All imaging procedures were performed under general anesthesia (see Supplementary Table S3).

<u>*Acute PET/MR imaging and analysis:*</u> Sheep were subjected to a 5 min $[^{15}O]H_2O$-PET scan, starting with a bolus injection of 1000 MBq of the tracer into the jugular vein. Blood samples were manually withdrawn at predefined time-points from a femoral artery during PET imaging for calculating the arterial input function. Simultaneously recorded anatomical and functional MR sequences included T1 3D MPRAGE (3D Magnetization Prepared Rapid Acquisition GRE; scan direction; sagittal; voxel size: 0.7x0.7x0.7 mm; acquisition matrix: 256x256; TE/TR: 2.66/1900 ms; number of averages: 2; flip angle: 9°) and T2 TSE (turbo spin echography; scan direction; coronal; voxel size: 0.39x0.39x1.48 mm; acquisition matrix: 384x307; TE/TR: 92/8630 ms; number of averages: 6; flip angle: 110°).

To delineate infarcted brain tissue, volumes of interest (VOIs) were drawn semi-manually and threshold-based on T2-weighted TSE-MR images for each animal individually, and then transferred to the corresponding and simultaneously acquired $[^{15}O]H_2O$-PET dataset. Absolute quantitative cerebral blood flow (CBF) maps were obtained voxelwise using the dedicated PMOD® software with the implemented CBF-calculation method (1-tissue-compartment model [5] and arterial input function. Voxel-wise identification of perfusion deficits independent of an absolute threshold as created for other species was performed due to partially suspect (increased) absolute CBF values, potentially arising as a result of isoflurane anesthesia during the imaging procedure. Therefore, VOIs were used for calculation of relative CBF (rCBF; %) referencing to corresponding areas in the contralateral hemisphere, and defined as the partial CBF of the ipsi- versus the contralateral hemisphere.

<u>*Follow-up imaging and analysis:*</u> The following sequences were applied using a SENSE-COIL-Flex M (Philips): T1 3D (scan direction: coronal; voxel size 0.39x0.39x0.98 mm; acquisition matrix: 256x228; TE/TR: 4.78/25 ms; flip angle: 30°) and T2 TSE (scan direction: coronal; voxel size: 0.23x0.23x2.00 mm; acquisition matrix: 256 205; TE/TR: 100/2315 ms; flip angel: 90°). Image processing was performed using MatlabR2010 (Mathworks, Germany) and SPM8 (Statistical Parametric Mapping, FIL Group, Wellcome Trust Center for Neuroimaging, London, UK). Initially, all T1 and T2 TSE weighted images were spatially

reoriented as defined by an ovine standard template [6], followed by reslicing to an isotropic voxel size of 0.5 mm. All data were coregistered to the ovine standard template and subsequently segmented using the ‘unified segmentation’ algorithm (MRF: 0.1, Sampling distance: 2) of SPM8 (‘New Segmentation’) [7]. Finally, the tissue and lesion mask volumes were calculated using MatLab2010 and SPM8.

Statistical analyses: The statistical evaluation was performed using SPSS 23.0 (SPSS Inc., Chicago Illinois, USA). Normal data distribution was confirmed using the Kolmogorov-Smirnov test (including Lilliefors test). Repeated measures analysis of variances (ANOVA) was used for comparison among measurement times. Simple contrasts were used to compare values after pMCAO with values prior to pMCAO. P-values <0.05 were considered statistically significant and $0.05 \leq p < 0.10$ as a tendency. Pearson’s correlation coefficient ($p<0.05$) was calculated to investigate a potential relationship among between APPs and IgA-, IgM- and IgG-anti-*E. coli* antibody concentrations, as well as between blood parameters and imaging parameters. Data are expressed as mean and standard deviation unless otherwise indicated.

Supplementary Figure

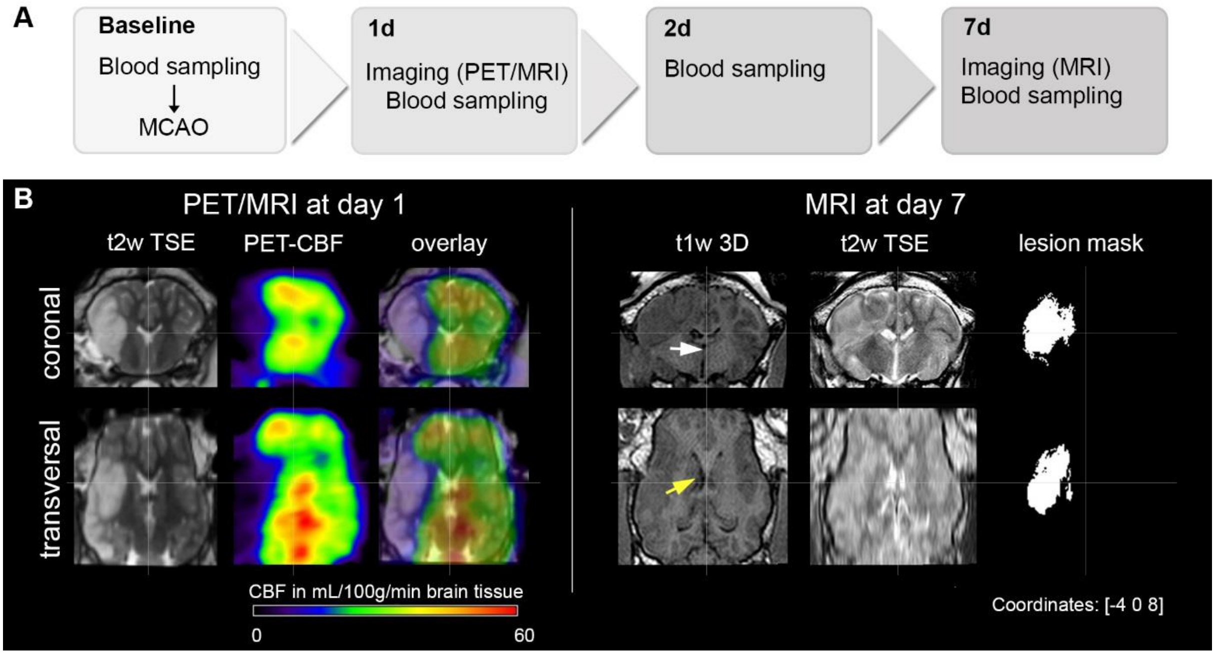

SF1: Study design and ischemic stroke impact. (A) Study design: multimodal 3T PET/MRI was used to confirm cerebral ischemia on day 1. A follow-up MRI (1.5T Philips) at day 7 was used to assess lesion configuration. Blood sampling was performed prior to permanent middle cerebral artery occlusion and at days 1, 2, and 7 thereafter. (B) Ischemic lesion after 1 day (left) and 7 days (right): The circumscript, hypertense area in t2w TSE and corresponding cerebral blood flow (CBF) values below 12 mL/100g/min indicate the ischemic lesion [5]). At day 7, the demarcated lesion appeared non-homogeneously in t2w TSE [8-9], potentially due to high resorption activities and necrotic remnants of brain tissue, the so called “fogging” effect [9]. Both, t1w and t2w TSE sequences were used to assess the infarct size [8-9]. Moreover, ipsilateral ventricle volume is increased (blue arrow) while a slight midline shift (white arrow) indicates a space-consuming edema.

Supplementary Tables

Table S1 Blood count with differential blood count results prior to surgery

Parameter	Value (mean±sd / quartile I-III)	Normal range
Leukocytes (G/l)	6.1 ± 2.1 / 4.8-7.3	5-11
Erythrocytes (T/l)	8.9±0.7 / 8.5-9.5	7-13
Thrombocytes (G/l)	512±212.8 / 309-636	280-650
Hemoglobin (mmol/l)	6.5±0.6 / 6.1-6.8	5.6-9.3
Hemtaocrit (l/l)	0.32±0.06 / 0.27-0.36	0.27-0.40
MCV (fl)	36.0±8.2 / 29.2-43.2	28-40
MCH (fmol)	0.74±0.06 / 0.71-0.78	0.6-0.7
MCHC (mmol/l)	21.08±3.81 / 18.58-24.47	19-23
Lymphocytes (%)	57.6±8.8 / 52.3-63.8	40-75
Monocytes (%)	2.3±1.6 / 1.0-4.0	0-6
Basophils (%)	0.2±0.4 / 0.0-0.0	0-3
Eosinophils (%)	3.4±3.5 / 1.0-5.3	0-10
Neutrophils (immature) (%)	0±0.0 / 0-0	0-2
Neutrophils (mature) (%)	36.5±8.1 / 30.0-39.5	10-50

sd – standard deviation; G/l – gigaparticle/liter; T/l – teraparticle/liter; f-femto; MCV – mean corpuscular volume; MCH – mean corpuscular hemoglobin; MCHC – mean corpuscular hemoglobin concentration.

Table S2 Clinical parameters prior to surgery

Parameter	Value (mean±sd / quantile I-III)	Normal range
Heart rate (bpm)	105±26.8/<br>83-118	70-140
Respiration frequency (min^{-1})	50±17.4/<br>41-60	20-100
Rectal temperature (°C)	38.9±0.3/<br>38.6-39.1	38-40

sd – standard deviation.

Table S3 Performed anesthesia and medication during surgical procedure and recovery period in all animals.

Purpose	Medication	Applied during
Anesthesia premedication	Intramuscular (i.m.) injection of 0.6 mg/kg midazolam (Ratiopharm, Ulm, Germany)	Surgery and imaging
Anesthesia induction and intubation	Intravenous injection of 6 mg/kg propofol (2%, Fresenius Kabi, Bad Homburg, Germany)	Surgery and imaging
General anesthesia I (injection)	Intravenous triple injection of:<br>6-10 mg/kg/h 2 % propofol and 6 mg/kg/h ketamine 0.3 mg/kg/h-midazolam (Ratiopharm, Germany)	Surgery and imaging
General anesthesia II (inhalation)	2% isoflurane (Abbvie, Germany) by controlled ventilation (40% O_2, 2 L/min oxygen flow)	pMCAO
Antibiosis and analgesia	Flunixin-meglumin 2.2 mg/kg/d, i.m. (Flunidol, *Cp-Pharm, Germany*)<br>Butorphanol 0.005 mg/kg, i.m. (Butorgesic, *Cp Pharm, Germany*)<br>Enrofloxacin (5 mg/kg, i.m.) (Baytril 5%, *Bayer Germany*)	Surgery + 3d post-surgery<br>Surgery day (every 4h)<br>Surgery + 5d post-surgery

d-days; pMCAO-permanent middle cerebral artery occlusion.

Supplementary Table S4 Imaging findings in the lesion brain following pMCAO.

Parameter	Value (mean±sd)
TSE defect volume - 1d	18±8 mL
TSE defect volume - 7d	18±8 mL
Total brain tissue volume - 7d (without lesion)	86±3 mL
Ischemic lesion - 7d	21±0.6 % of brain tissue

pMCAO - permanent middle cerebral artery occlusion; d – days; ischemic lesion –relative cerebral blood flow deficit, defined as the partial CBF deficit of the ipsi- to contralateral hemisphere

Supplementary Table S5 Correlations between APPs and IP markers 1d pMCAO

**Blood parameters**	**CRP** R (p-value)	**LBP** R (p-value)
IgG-anti-*E.coli*	0.167 (0.667)	0.176 (0.651)
IgM-anti-*E.coli*	-0.312 (0.667)	0.096 (0.806)
IgA-anti- *E.coli*	0.540 (0.133)	**0.700* (0.036)**

APPs - acute phase proteins; IP - intestinal permeability; pMCAO - permanent middle cerebral artery occlusion; CRP - C-reactive protein; LBP - lipopolysaccharide binding protein; IgM-, IgA-and IgG-anti-*E. coli* antibody; R - Pearson’s correlation coefficient; p - significance level, ***** indicates p < 0.05.

Supplementary Table S6 Correlations between APPs and IP markers 2d pMCAO

**Blood parameter**	**CRP** R (p-value)	**LBP** R (p-value)
IgG-anti-*E.coli*	-0.049 (0.917)	0.352 (0.393)
IgM-anti-*E.coli*	-0.448 (0.313)	0.138 (0.745)
IgA-anti-*E.coli*	0.344 (0.449)	-0.214 (0.611)

APPs - acute phase proteins; IP - intestinal permeability; pMCAO - permanent middle; cerebral artery occlusion; CRP - C-reactive protein; LBP - lipopolysaccharide binding protein; IgM-, IgA-and IgG-anti-*E. coli* antibody; R - Pearson’s correlation coefficient ; p - significance level.

Supplementary Table S7 Correlations between APPs and IP markers 7d pMCAO

**Blood parameter**	**CRP** R (p-value)	**LBP** R (p-value)
IgG-anti-*E.coli*	-0.265 (0.565)	0.384 (0.396)
IgM-anti-*E.coli*	-0.655 (0.110)	0.361 (0.426)
IgA-anti- *E.coli*	-0.020 (0.966)	-0.592 (0.161)

APPs - acute phase proteins; IP - intestinal permeability; pMCAO - permanent middle; cerebral artery occlusion; CRP - C-reactive protein; LBP - lipopolysaccharide binding protein; IgM-, IgA-and IgG-anti-*E. coli* antibody; R - Pearson's correlation coefficient ; p - significance level.

Table S8 Correlations between infarct characteristics and blood parameters 1d post pMCAO

**Blood parameter**	**rCBF** R (p-value)	**Infarct volume** R (p-value)
CRP	-0.546 (0.205)	-0.434 (0.282)
LBP	0.223 (0.630)	0.057 (0.893)
IgG-anti-*E.coli*	0.278 (0.546)	-0.156 (0.712)
IgM-anti-*E.coli*	**0.927* (0.003)**	-0.516 (0.190)
IgA-anti-*E.coli*	-0.283 (0.538)	-0.502 (0.205)

pMCAO - permanent middle cerebral artery occlusion; CRP - C-reactive protein; LBP - lipopolysaccharide binding Protein; IgM-, IgA-and IgG-anti-*E. coli* antibody; rCBF - relative cerebral blood flow, defined as the partial CBF of the ipsi- to contralateral hemisphere; Infarct volume-TSE defect volume [cm3]; R-Pearson's correlation coefficient; p - significance level, ***** indicates $p < 0.05$.

Supplementary References

1. Boltze J, Förschler A, Nitzsche B, Waldmin D, Hoffmann A, Boltze CM, Dreyer AY, Goldammer A, Reischauer A, Härtig W, Geiger KD, Barthel H, Emmrich F, Gille U. (2008): Permanent middle cerebral artery occlusion in sheep: a novel large animal model of focal cerebral ischemia. J Cereb Blood Flow Metab. 28(12):1951-1964.

2. Schroedl W, Fuerll B, Reinhold P, Krueger, M, Schuett C. (2001): A novel acute phase marker in cattle: lipopolysaccharide binding protein (LBP). J Endotoxin Res. 7(1):49-52.

3. Schroedl W, Jaekel L, Krueger M. (2003): C-reactive protein and antibacterial activity in blood plasma of colostrum-fed calves and the effect of lactulose. J Dairy Sci. doi: 10.3168/jds.S0022-0302(03)73933-2.

4. Sarikaputi, M.; Morimatsu, M.; Syuto, B.; Saito, M.; Naiki, M. (1991): A new purification procedure for bovine C-reactive protein and serum amyloid P component. In: The International journal of biochemistry 23 (10), S. 1137–1142.

5. Alpert NM, Eriksson L, Chang JY, Bergstrom M, Litton JE, Correira JA. (1984) Strategy for the measurement of regional cerebral blood flow using short-lived tracers and emission tomography. J Cereb Blood Flow Metab. 4(1):28-34.

6. Nitzsche B, Frey S, Collins LD, Seeger J, Lobsien D, Dreyer AY, Kirsten H, Stoffel MH, Fonov VS, Boltze J. (2015): A stereotaxic, population-averaged T1w ovine brain atlas including cerebral morphology and tissue volumes. Front. Neuroanat. doi: 10.3389/fnana.2015.00069.

7. Ashburner J, Friston KJ. (2005): Unified segmentation. In: NeuroImage. 26(3):839-851.

8 Boltze J, Nitzsche B, Geiger KD, Schoon HA (2011): Histopathological Investigation of Different MCAO Modalities and Impact of Autologous Bone Marrow Mononuclear Cell Administration in an Ovine Stroke Model. Transl Stroke Res. 2(3):279-93.

9. Wagner DC, Deten A, Härtig W, Boltze J, Kranz A. (2012): Changes in T2 relaxation time after stroke reflect clearing processes. Neuroimage. 61(4):780-5.
